# Impact of a resilience and wellbeing program: A longitudinal cohort study of student dietitians

**DOI:** 10.1111/nhs.12957

**Published:** 2022-06-24

**Authors:** Lynda J. Ross, Lana J. Mitchell, Emily C. Williams, Patrick J. Lynch, Jonathan P. Munro, Lauren T. Williams

**Affiliations:** ^1^ School of Exercise and Nutrition Sciences, Faculty of Health Queensland University of Technology (QUT) Brisbane Queensland Australia; ^2^ School of Health Sciences and Social Work Griffith University Gold Coast Queensland Australia; ^3^ Student Health, Counselling & Wellbeing Griffith University Gold Coast Queensland Australia; ^4^ School of Applied Psychology Griffith University Gold Coast Queensland Australia

**Keywords:** dietitian, education, mental health, practicum, resilience, wellbeing

## Abstract

In response to growing evidence that student healthcare professionals find professional practicum stressful and that it negatively affects their mental health, a six‐session psychoeducation Resilience and Wellbeing Program was implemented by a professional counselor in Year 3 of the Bachelor of Nutrition and Dietetics at Griffith University, Australia. The aim of this study was to evaluate student dietitians' perceptions of whether the program improved their ability to cope with practicum stressors. The study used a longitudinal cohort design, with students completing surveys at three time points: before and after the program and after the final practicum. The study was completed with two cohorts of students between 2018 and 2020 (n = 111). Most respondents (95%) found their professional practicum to be stressful or challenging on at least some occasions, mostly due to constantly being assessed (56%), finances (40%), and being away from usual supports (38%). Almost all students rated the program as having some value (99%), with the content about stress and self‐care the most highly rated. Qualitative comments revealed the program helped students to manage stress by prioritizing their personal needs. Students used stress management skills during the practicum to achieve balance in their lives, despite pandemic conditions.

**Key points**

- Students valued a Resilience and Wellbeing Program in the year prior to the professional practicum.
- A Resilience and Wellbeing Program helped students recognize that managing stress and prioritizing self‐care while on practicum was important for their mental health and well‐being.
- Following the Resilience and Wellbeing Program, students demonstrated increased resilience and stress management skills to achieve a growth mindset and balance in their lives.

## 1 INTRODUCTION

Taking steps to ensure that future healthcare professionals are workforce ready is a critical step for advancing health professions (Edward et al., 2017) and the health of the population (World Health Organization, 2006). Professional practicum placements play an important role in the preparation process. In Australia, the national body that accredits dietitians, Dietitians Australia (formerly Dietitians Association of Australia), requires practitioners to graduate from an accredited course that includes 100 days of professional practicum (Dietitians Association of Australia, 2015). By the end of the practicum, students are required to demonstrate competence for independent practice as measured against national competency standards (Dietitians Association of Australia, 2015, 2017). The practicum is an immersive experience that provides students with an opportunity to integrate theory and practice and develop the skills and attributes of a healthcare professional (Markwell et al., 2021). Students' success on their practicum is influenced by the quality of their teaching and assessment, adequate preparation for practice (Ottrey et al., 2021), and their own personal attributes (Maher et al., 2015).

Dietetics students complete a significant proportion of their practicum in the hospital setting, an acknowledged stressful environment even for the experienced healthcare professional (Thapa et al., 2021). Healthcare students face the emotional and cognitive burden of caregiving roles (Sheu et al., 2002) with the additional stressors of being in an unfamiliar environment (Maher et al., 2015), being subjected to academic pressures (Bhurtun et al., 2019), financial pressures (Patten & Vaterlaus, 2021) and working toward their own professionalization (Murphy et al., 2009). Students need to be provided with strategies to manage their own mental health and well‐being (Ruiz‐Aranda et al., 2014) and strategies to minimize the impact of stressful situations through workload management, work–life balance, and metacognitive coping processes (Huey & Palaganas, 2020). Although there is good evidence for a relationship between well‐being and resilience in the workplace, the concept is not adequately addressed in health professional education (Low et al., 2019).

The ability to cope successfully and to thrive within the healthcare environment is increasingly viewed as a critical graduate capability (Tomlinson, 2017), with well‐being and resilience both considered as factors for success. Well‐being and resilience are incorporated into the competency standards for Australian dietitians: graduates must accept responsibility for and manage, implement, and evaluate their own personal health and well‐being (standard 1.1.3), and demonstrate flexibility, adaptability, resilience, and the ability to manage their own emotions (standard 1.1.7) (Dietitians Australia, 2021). However, the need for dietitians to receive additional training in resilience has been identified (Blair et al., 2021). Well‐being can be defined as the presence of favorable emotions, including happiness and contentment (Medvedev & Landhuis, 2018). However, there are differences of opinion in how resilience is defined. Resilience has been described as either a capability in the face of adversity or difficult circumstances or adaptation and rebounding following an adverse event (Brewer et al., 2019; Huey & Palaganas, 2020). Resilience can also be influenced by individual, environmental, and organizational factors, as well as by how an individual interacts with their circumstances (Brewer et al., 2019; Huey & Palaganas, 2020). Individual factors include internal locus of control, positive outlook, self‐determination, and being more adaptable to change (Huey & Palaganas, 2020; McAllister & McKinnon, 2009). Resilience and well‐being are positively influenced by supportive relationships and a sense of belonging (Huey & Palaganas, 2020).

Brewer and colleagues suggest resilience needs to be learned (Brewer et al., 2019), and that healthcare supervisors should not expect students to inherently display this skill. Studies in the health professions have focused on elucidating the characteristics of resilience and identifying strategies that can build resilience (Low et al., 2019). In a scoping review of strategies used in higher education to teach resilience, Low et al. (2019) identified ways to build resilience in healthcare students (medical, nursing, social work, and psychology), including reframing stress and implementing self‐care, mindfulness, and meditation practices. Stallman (2011) evaluated the feasibility of a resilience‐building intervention in 247 university students. Through surveys and personal reflective journal entries the study identified important considerations for designing educational programs to enhance resilience, including the need for students to identify their own goals and work toward personal intervention strategies that are practical and address multiple risk factors (Stallman, 2011). However, it is unclear if any interventions targeting resilience have been conducted with dietetics students.

In response to concerns about a high practicum failure rate in the bachelor of nutrition & dietetics (BND) at Griffith University, Australia, a practicum preparation module was introduced into the final year curriculum. Students completed the preparation module immediately prior to their practicum. Evaluation demonstrated the preparation module increased student confidence before commencing the practicum (Ross et al., 2016, 2017). However, a subsequent critical incident study of practicum experiences showed students continued to find placement in the hospital environment stressful, with negative mental health impacts. To address this concern, a psychoeducation program was developed to support student resilience and well‐being and was introduced into the BND in the year prior to the practicum. Psychoeducation embraces several complementary theories and models of clinical practice. These include ecological systems theory, cognitive behavioral theory (CBT), learning theory, group practice models, stress and coping models, social support models, and narrative approaches (McFarlane et al., 2003). In contrast to psychosocial interventions that use traditional models to treat pathology, illness, liability, and dysfunction (Lukens & McFarlane, 2006), psychoeducation models reflect a paradigm shift to a more holistic and competence‐based approach to teaching and learning (Lukens & McFarlane, 2006).

The aim of this study was to evaluate the impact of a Resilience and Wellbeing Program by surveying students before and after and again after their practicum to assess its contribution to building resilience against practicum stress.

## 2 METHODS

### 2.1 Study design

A longitudinal cohort study design was used to evaluate the Resilience and Wellbeing Program at Griffith University, Australia. Surveys were conducted with two cohorts of students enrolled in the undergraduate nutrition and dietetics course: students entering third year in 2018 and students entering third year in 2019. Participants were provided with information about the study and invited to complete the survey, with completion implying voluntary consent. Approval to conduct the study was gained from the Griffith University Human Research Ethics Committee (HREC 2014/826; 2019/250). Reporting used the Strengthening the Reporting of Observational Studies in Epidemiology (STROBE) checklist.

### 2.2 Setting

The university offers a 4‐year BND undergraduate course that includes 100 days of full‐time professional practicum in the final year. Practicum placements are in the key areas of medical nutrition therapy (50‐day placement in hospital‐based acute and nonacute care), community and public health nutrition (30‐day placement in community centers) and food service management (20‐day placement in hospital‐based food services). Students attend the practicum in pairs or small groups. In the medical nutrition therapy practicum, a peer‐assisted learning model is used to facilitate learning.

### 2.3 Participants

Participants for this research were students enrolled in third year of the BND course, either 2018 or 2019 (n = 111). They completed the program in the third year and completed their final practicum and graduated in the following year, either 2019 or 2020 (*n* = 105). These two participant cohorts are referred to in this paper as G2019 (Year 3, 2018 *n* = 55; Year 4, 2019 *n* = 57) and G2020 (Year 3, 2019 *n* = 56; Year 4, 2020 *n* = 48). The G2020 cohort completed their practicum in an environment that was highly influenced by the COVID‐19 pandemic; however, placements were able to be completed.

### 2.4 The Resilience and Wellbeing Program

**FIGURE 1:**
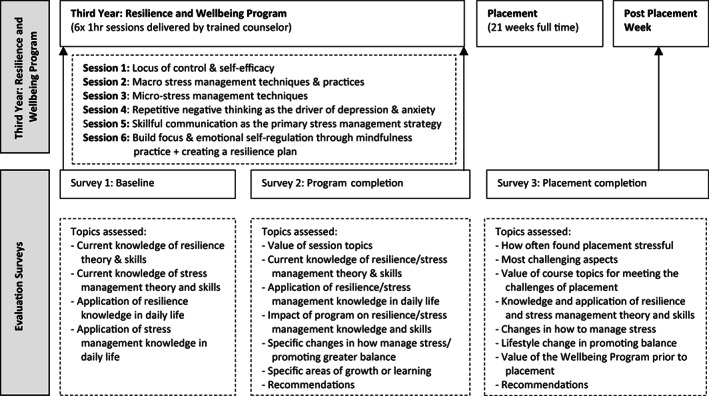
Overview of the study design showing the 6‐week Resilience and Wellbeing Program delivered in Year 3 of the bachelor of nutrition & dietetics in the trimester before attending final year placement in Year 4

The program was embedded into BND coursework requirements in the final trimester of the third year to educate students on theory and skills related to resilience and stress management in preparation for the practicum period. The 6‐hour program was developed by the Counselling and Wellbeing Unit of Griffith University and delivered by a qualified student health counselor. The intervention was conducted as hourly sessions over 6 weeks. The content included topics related to resilience, stress management, communication, self‐efficacy, and self‐regulation (outlined in Figure 1).

Sessions included didactic delivery, videos, TED talks, individual reflections, and group discussions. The program explicitly taught students about the importance of self‐care, resilience, and coping strategies (Beddoe et al., 2013; Kreitzer & Klatt, 2017). It encouraged students to identify and implement self‐care strategies (McDonald et al., 2013; Skovholt & Trotter‐Mathison, 2014) and focus on positive reframing as a specific coping technique (Steinhardt & Dolbier, 2008).

The program's content was developed following a review of relevant literature, a review of resilience‐based courses offered by health facilities and universities internationally, and feedback from students who had previously attended counseling services. Based on evidence that successful educational strategies require learning activities that are underpinned by theoretical frameworks (O'Shea et al., 2021), ecological systems theory was used for assessing and helping student groups to consider the content. Students were encouraged to examine likely experiences on practicum in relation to other existing systems in their lives (i.e., partners, family, school, workplace). The purpose of a therapy group is typically for education, training, or support and psychoeducational groups provide opportunities for participants to understand and solve problems that affect their functioning and to gain skills for daily life (Brown, 2018). Such models reduce isolation and provide a forum for both recognizing and normalizing experience and response patterns among participants. CBT techniques such as problem‐solving and role‐play enhanced the presentation of didactic material by allowing participants to rehearse and review new information and skills in a safe setting (Lukens & McFarlane, 2006). These activities were amplified through specific attention to developing stress management and other coping techniques (McFarlane, 2004). Narrative models, in which students were encouraged to recount stories related to the circumstances at hand, were used to help them recognize personal strengths and resources (resilience) and generate possibilities for action and growth (White & Waters, 2015).

### 2.5 Survey design

The surveys were designed to evaluate the program by the researchers, who were a qualified student health counselor at the university and academics specializing in psychology or nutrition and dietetics. Items were designed to assess the program through before and after self‐ratings of importance and topic‐related knowledge of theory and the application of knowledge in relation to resilience and stress, the two key components of the program. No pretesting was conducted. Likert scales were largely used owing to their reliability and validity and wide use in psychological, social science, and medical education research and to provide nuance and insight into participant opinions (Joshi et al., 2015). Open‐ended free text questions were also included to further elucidate student opinion and practice of learned theory and strategies in their daily lives.

### 2.6 Data collection

Survey data were collected from participants at three time points, as shown in Figure 1. Survey 1 was completed via hard copy at the start of Session 1 of the program (in both 2018 and 2019). Survey 2 was completed at the end of Session 6. Survey 3 was completed online via Survey Monkey (Momentive Inc., San Mateo, CA, USA, https://www.surveymonkey.com) when participants had completed their final practicum and returned to university for final assessment (2019 and 2020). Student responses were not linked over time.

The surveys were designed to examine students' knowledge and application of stress management theory and skills, and their knowledge and application of resilience theory and skills. Survey 1 comprised 11 items (seven Likert scale and four open‐ended, free text questions). Survey 2 comprised 13 items (nine Likert scale and four open‐ended, free text questions). Survey 3 comprised 13 items (seven Likert scale, one multiple‐response, and five open‐ended, free text questions). Both Surveys 2 and 3 included items asking students to rate the perceived value of individual topics and to rate the overall program on a seven‐point Likert scale (from (1) not valuable to (7) highly valuable). Knowledge and application of resilience and stress management theory and skills were rated on a seven‐point Likert scale (from (1) low to (7) high) on all three surveys. Survey 3 also contained items asking students to rate how stressful or challenging they found practicum (rarely, occasionally, frequently, almost always) and to indicate the most challenging aspects (from a list of potential challenges). Open‐ended items asked about specific changes to lifestyle behaviors, the management of stress during practicum, and specific areas of growth and learning.

### 2.7 Data analysis

Survey data in hard copy surveys were manually entered and electronic data in Survey Monkey were exported to Microsoft Excel (Microsoft 365, Version 2108 L) and IBM SPSS Statistics (Version 25). Summary statistics were used to describe Likert‐scale data. Because of the small sample size, the Shapiro–Wilk test was performed and showed a nonnormal distribution. However, the mean, median, and mode, all valid measures of central tendency, were found to be not appreciably different and there were no outliers (Sullivan & Artino Jr, 2013). Therefore, the mean and standard deviation (SD) were used in tabulations to allow for easier comparison by future studies. The results were analyzed through a series of descriptive and frequency comparisons. Independent *t*‐tests were completed to evaluate differences between the two student cohorts (G2019 and G2020). Statistical significance was set at *p* < 0.05. Qualitative responses from open‐ended items were analyzed using thematic analysis as outlined by Braun and Clarke (2006). To address reflexivity, five members of the research team contributed across different stages of analysis: (1) familiarizing with the data (LM and LR), (2) generating initial codes (LM), (3) searching for themes (LM and LR), (4) reviewing themes (LM, EW, PL), (5) defining and naming themes LM and LW), and (6) producing the report (Braun & Clarke, 2006). All members of the research team agreed on the themes identified. Qualitative exploration of the themes was conducted, using contextual excerpts to support quantitative findings, and discussed with the research team.

## 3 RESULTS

Survey 1 was completed by 84 Year 3 students (76% of the combined cohorts) (G2019: *n* = 36, 65%; G2020: *n* = 48, 86%). Survey 2 was completed by 76 Year 3 students (68% of the combined cohorts) (G2019: *n* = 35, 64%; G2020: *n* = 41, 73%). Survey 3 was completed by 94 Year 4 students (90% of the combined cohorts) (G2019: *n* = 48, 84%; G2020: *n* = 46, 96%).

**FIGURE 2:**
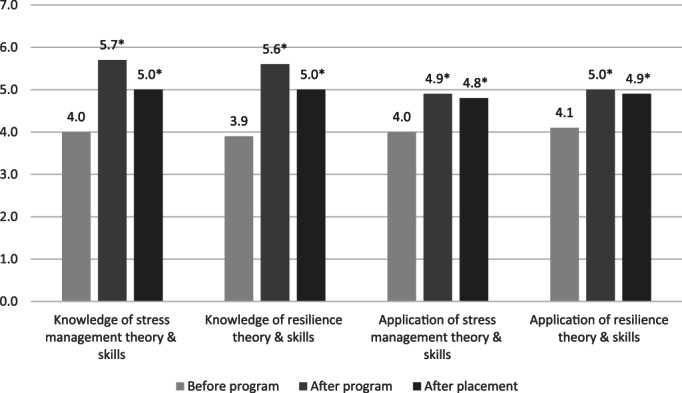
Mean ratings of students' perceived knowledge and application of theory and skills before (Survey 1, *n* = 84) and after (survey 2, *n* = 74) the Resilience and Wellbeing Program, and after placement (Survey 3, *n* = 94). * significantly different from Survey 1, *p* < 0.01

At each survey time point, students were asked to rate their knowledge of theory and skills in relation to stress management and resilience as well as the application of this knowledge in their daily lives. Figure 2 shows the mean Likert‐score values (1 = low; 7 = high knowledge or application) for combined data (G2019 and G2020) before and after the program and after practicum. There were no significant differences in scored ratings between cohorts at Survey 1. Knowledge ratings for stress management and resilience theory and skills were significantly higher after the program (Survey 2) compared to before the start of the program (Survey 1) (*p* < 0.001), with a small difference between cohorts for “Knowledge of Resilience Theory & Skills” (G2019 5.9 versus G2020 5.4, *p* = 0.03). Mean knowledge ratings after the program (Survey 2) and after practicum (Survey 3) were significantly higher than before the program (Survey 1, *p* < 0.001), with no significant differences between the cohorts. However, Survey 3 ratings for resilience knowledge were significantly lower than at Survey 2 (*p* = 0.039). Ratings of the application of stress management and of resilience theory and skills were significantly higher at Survey 2 compared to Survey 1 (*p* < 0.001), with no significant differences between cohorts. Students maintained these significantly higher ratings after practicum (Survey 3) compared to Survey 1, with no significant differences between cohorts.

**FIGURE 3:**
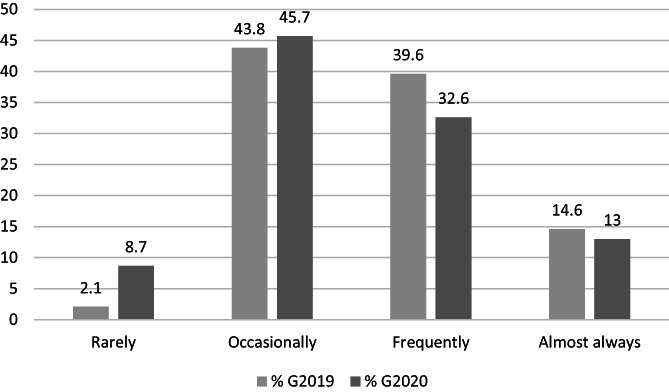
Percentage frequency of student perceptions of how often they found placement stressful/challenging at completion of final placement in Year 4 (*n* = 94)

Figure 3 shows the frequency of student responses on the postpracticum survey when rating the question *“How often did you find placement stressful/challenging?”* In total, 95% (*n* = 89/94) of students rated practicum as stressful/challenging on at least some occasions, and 50% (*n* = 47/94) found this to be the case frequently or almost always. Results were similar between cohorts, with no statistically significant between‐cohort differences.

**FIGURE 4:**
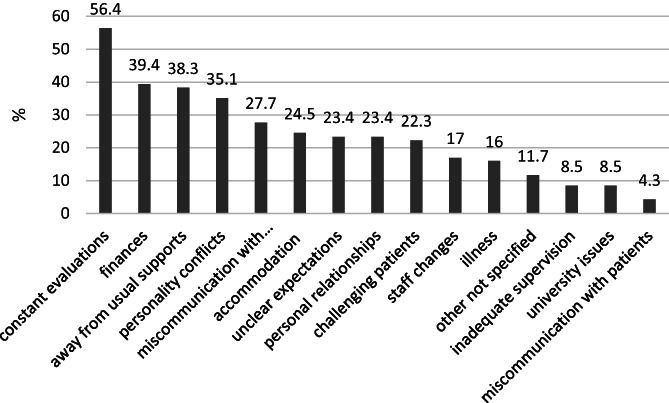
Percentage frequency of student perceptions of the most challenging aspects of professional placement (*n* = 98)

Figure 4 represents the frequency of students' selections from a list of possible challenging aspects of practicum on Survey 3. Students could select as many factors as they perceived as relevant, and the number of responses per individual student ranged from one to seven (ignoring outliers). The median number of selections per individual student was three (mean 3.6 (range 3.4–4.2) across all groups (raw data not shown). The four most challenging aspects of practicum were: “constant assessment” (*n* = 55/98, 56% of responses), “finances” (*n* = 39/98, 40%), “being away from usual supports” (*n* = 37/98, 38%), and “personality conflicts” (*n* = 34/98, 35%). Assessment‐related aspects, including “constant evaluation with assessment,” “unclear expectations with assessment,” and “insufficient or inadequate supervision with assessment,” were reported by 66% (*n* = 65/98) of students. One or more responses to relationship‐related aspects were common for “personality conflicts” (*n* = 34/98, 35%), “personal relationships” (*n* = 23/98, 23%), “staff changes” (*n* = 17/98, 17%,), and “inadequate supervision” (*n* = 9/98, 9%). The “other not specified” category, selected by 11.7% (*n* = 12/98) of students, included factors such as working in groups/peer‐assisted learning, other issues with supervisors/clinical educators, working from home due to the COVID‐19 pandemic, completing unfinalized assessment from previous practicum, confidence, and managing work–life balance.

**FIGURE 5:**
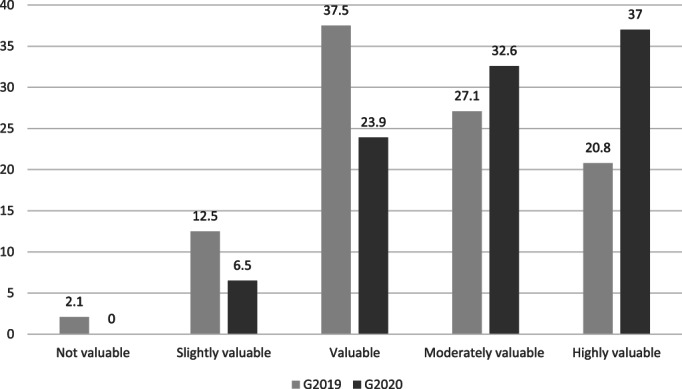
Percentage frequency of students' perceptions of how valuable it is to have a Resilience and Wellbeing Program before placement, captured at completion of final year placement (*n* = 94)

Figure 5 shows the frequency of student ratings on the post‐practicum survey in response to the question **“** *On reflection, how valuable is it to have a Resilience and Wellbeing Program prior to placement?”* In total, 99% (*n* = 93/94) of students found some value in the overall program and a majority (58.5%) found it to be moderately or highly valuable. There were no statistically significant differences between cohorts. Participants also rated individual session topics (Survey 2, data not shown) as valuable, with mean rankings ranging from 5.6–6.4 out of 7. “Stress and self‐care” was the most highly rated individual session (mean 5.9; mode 7). However, after the practicum, the perceived value of individual sessions dropped slightly, with means ranging from 4.6–5.8 out of 7.

Open‐ended responses on whether students perceived the program had “*led to any specific changes in how you manage stress*” and “*promoted greater balance*” were organized into three themes and 10 subthemes (Table 1).

**TABLE 1:** Themes arising from student perceptions of behavior changes in response to a Resilience and Wellbeing Program

Theme	Subtheme	Indicative quote
Recognizing the impact of stress and importance of managing stress	1a. Recognizing role and impact of stress and emotions	*“Just being able to recognize it before it becomes an issue.” (G2019, P15)* *“I am more conscious of my triggers and have some specified self‐care measures that I use when I am feeling stressed.” (G2019, P25)* *“Helped me to understand how to use stress to help me.” (G2020, P61)*
	1b. Acknowledging the importance of self‐care	*“Greater self‐care importance.” (G2020, P61)* *“Knowing what my self‐care is.” (G2019, P21)* *“Appreciation for the (self‐care) I already do.” (G2019, P43)*
2 Prioritizing personal needs	2a. Prioritizing physiological needs (exercise, meal, sleep)	*“I have always ensured to maintain a healthy lifestyle and exercise regularly but the course helped to highlight the importance of allocating time to exercise and cook healthy meals.” (G2020, P52)* *“I now meal prep and it has helped make sure I have enough time to eat a healthy meal for lunch each day.” (G2019, P48)*
	2b. Prioritizing mental health needs (self‐care, meditation/ breathwork)	*“Prioritizing my mental health when it becomes overwhelming and not using uni as an excuse to not care for myself or prioritize my mental health… have some specified self‐care measures that I use when I am feeling stressed.” (G2019, P25)* *“More focus on deep breathing, self‐care, and acknowledging emotions.” (G2020, P65)* *“Yes, I ensure that I acknowledge stress and act on this by … doing some meditation.” (G2020, P91)*
	2c. Seeking support and connection	*“Seek more support when needed.” (G2020, P50)* *“Talking to family or friends.” (G2020, P91)* *“Check‐ins from supervisors etc reminding about self‐care (whilst repetitive) was really helpful. I felt supported knowing they expected us to have down time and not constantly be up‐skilling.” (G2019)*
	2d. Winding down and ensuring adequate leisure time	*“Ensuring I de‐stress and participate regularly in leisure activities/things that make me calm and present.” (G2020, P60)* *“At the end of the day I spend about 30 mins – 1 hour to really wind down then get up and continue my night.” (G2020, P71)* *“I take breaks away from study in the evenings and weekends.” (G2019)*
3 Growth mindset and achieving balance	3a. Recognizing locus of control	*“I focus on what I can control/change and try to let go of the rest. I understand nerves and anxiety are normal and natural – they do not mean I will not perform well.” (G2020, P65)* *“I feel like I remain more calm and accept things as they are and deal with it instead of over stressing.” (G2019, P34)* *“Overcoming repetitive negative thinking.” (G2020, P54)* *“At the beginning of the day I contemplate on my stress levels upon waking up then really think about why am I stressed and decide whether or not I really need to stress about it.” (G2020, P71)*
	3b. Building resilience	*“Course was very helpful and I use techniques surrounding building resilience most days (taking the personal aspect out of situations).” (G2020, P57)*
	3c. Achieving work–life balance	*“More of a work–life balance rather than just prioritizing work and uni only.” (G2019, P15)* *“Leaving work at work and enjoying free time.” (G2019, P21)* *“Realizing there is more to life than just uni and doing things outside of that make me perform better.” (G2020, P67)* *“I felt like I had more time for ‘me’ as I had my weekends completely free as I did not work like I did in third year.” (G2020, P82)*
	3d. Using organizational skills to achieve balance	*“Making clear plans and goals.” (G2020, P85)* *“I stay organized to avoid stress.” (G2019, P12)* *“Better time management and organizational skills.” (G2019, P21)*

*Note*: G2019 *n* = 48, participated in the program in 2018; completed placement and graduation in 2019; G2020 *n* = 46, participated in the program in 2019; completed placement and graduation in 2020.

Abbreviation: P, participant.

The responses demonstrated how the program assisted students to manage and cope with stress during their practicum year. Students implemented lifestyle behavior changes by recognizing the impact of stress and the importance of managing stress (Theme 1), and by prioritizing their personal needs (Theme 2); they also experienced a growth mindset and achieved balance in their lives (Theme 3).

Theme 1 shows students were able to recognize the role and impact of stress and emotions. This was expressed by students' increased awareness of negative thinking, stress, and anxiety; development of stress management techniques; and building of emotional self‐regulation through mindfulness practice. Students reported identifying personal triggers and helpful behaviors, as well as understanding the important role stress plays in performance. The subtheme “Acknowledging the importance of self‐care” emphasizes students' use of personal self‐care strategies and their appreciation of the self‐care already being undertaken.

Theme 2 encompasses many of the stress management and coping strategies implemented by students. The subtheme “Prioritizing physiological and mental health needs” demonstrates that students were able to recognize the importance of actioning healthy lifestyle activities and that mental health care was often overlooked when under stress. Therefore, many students took deliberate steps for self‐care while on practicum. The subtheme “Seeking support and connection” emphasizes that students were able to acknowledge the importance of relationships across the range of people having influence in their lives, and the subtheme “Winding down and ensuring adequate leisure time” emphasizes how students were able to consider the importance of taking action to promote balance outside the practicum experience.

Theme 3 indicated that students perceived the program led to the development of a growth mindset that had a positive impact on the way they managed stress in their lives. The subthemes of “Recognizing locus of control” and “Building resilience” demonstrated that students had become more aware of their stress levels and were able to focus on what they could change. The subthemes of “Achieving work–life balance” and “Using organization skills to achieve balance” highlighted how proactive strategies, such as taking time away from practicum and implementing better time management, helped to reduce their stress levels.

## 4 DISCUSSION

This study provides evidence that student dietitians' perceptions of resilience increased following a low‐intensity well‐being intervention using a theory‐based psychoeducation model. The positive effects of the program were maintained during the students’ professional practicum, conducted the following year. Students reported the application of new knowledge and skills, which had positive impacts on their mental health, well‐being and everyday behavior. The findings of this study align with literature that suggests students need to be taught to develop attributes such as resilience and strategies for adapting to the challenges of stressful environments and situations (Low et al., 2019). Previous studies have indicated that effective educational interventions can improve resilience (Huey & Palaganas, 2020). Our results indicate that teaching student dietitians how to recognize and manage stress can promote positive behavior change and resilience during their time on practicum.

The students surveyed in this study acknowledged they found the practicum to be stressful and challenging, in line with existing literature (Maher et al., 2015). Many believed the most challenging aspect was constantly being assessed. This might be expected considering the academic pressures of the student experience, which involves being observed in the workplace with high stakes outcomes for the final stage of a 4‐year degree (Bhurtun et al., 2019), all while working in demanding and challenging clinical environments (Murphy et al., 2009). As students advance in their training and become more responsible for their learning and their patients, these pressures are likely to escalate (Epstein et al., 2019). For these students, added pressures included the emotional and financial burdens of being away from home and from usual supports and regular income, reported previously (Tucker et al., 2006), as well as recent uncertainties and health risks associated with a global pandemic. One cohort of student participants experienced practicum during the COVID‐19 pandemic, which may have added stressors. Interestingly, in free text responses, these students did not write about this aspect of practicum stressors.

Although perceived knowledge of theory and skills in relation to resilience and stress management increased significantly compared to baseline, ratings of resilience knowledge were higher immediately after the program (Survey 2) than later, after practicum completion (Survey 3). This suggests an effect of time between program delivery and the postpracticum survey (up to 1 year), with outcomes diminishing over time. However, another explanation could be the effect of hindsight about the practicum experience, which may have moderated students' earlier responses and requires further research. In comparison, when students were asked to rate the application of their new knowledge and skills into their everyday lives, high ratings immediately after the program were maintained post practicum. These results suggest that behavior change strategies to support facets of resilience and stress management were equally important and applicable over the long term, both in students' general lives as well as during the practicum.

Although practica form an essential component of health degrees, they have inherent stressors (Thapa et al., 2021). The main reason the program was introduced within the university's BND course was to prepare students to respond positively to the stress‐inducing challenges of the practicum. A range of theoretical frameworks and learning and teaching models were combined in a psychoeducation model to address students' mental health needs. As a result, students developed self‐awareness, were able to demonstrate competence in being responsible for their own health and well‐being, and were able to identify and implement self‐care strategies (Skovholt & Trotter‐Mathison, 2014). In the literature, enhancing attributes such as self‐awareness and personal competence are considered to be important for maintaining physical, mental, and emotional health throughout a health professional's career (Low et al., 2019). It was not surprising then, that students perceived attending a resilience and wellbeing program as valuable and rated relevant topics as highly valuable.

The free text responses to the survey showed how the program assisted students to manage and cope with stress in their lives and confirmed their understanding of the role of stress and emotion in the context of the practicum. The role of self‐reflection and mindfulness, especially from a strength‐based approach, appeared to be effective for building resilience by focusing on solutions and coping strategies (Low et al., 2019), including focusing on the positives and reframing (Steinhardt & Dolbier, 2008). The program's holistic and narrative approach to mental health and collaboration with others encouraged students to recognize their strengths and resilience and to prioritize their personal needs. Hence, students reported ways of addressing both their physiological and mental health needs as well as seeking connection and support from others. Previous studies have identified that being capable of self‐care is essential for promoting resilience, including identifying the need for self‐care, actively accessing self‐care activities, and making time (Huey & Palaganas, 2020). Students also learned to recognize their internal locus of control, a theoretical construct designed to assess a person's perceived control over their own behavior. Students reported actions that achieved balance in their lives and developed a sense of empowerment through a level of influence and control over events. Previous studies have identified that the ability to achieve work–life balance and being able to relax and engage in leisure time activities can build resilience and reduce burnout (Huey & Palaganas, 2020).

A number of limitations exist with this study. Data were collected from a portion of students from just two cohorts at the one university site, providing a small study group that may not be representative of other students at other universities. Although longitudinal data are important for establishing changes, the anonymous surveys were not linked to allow individual responses to be tracked over time, and there was no comparison group of students who did not receive the intervention. The use of validated tools for evaluating well‐being and resilience would also have been useful.

## 5 CONCLUSION

This longitudinal study shows that a low‐intensity psychoeducation intervention focused on building resilience in student dietitians was successful in assisting them to cope with the acknowledged stressors of the professional practicum. The program was founded on ecological and CBT theoretical frameworks and learning models to allow students to develop coping and stress management strategies and enable them to demonstrate competence in managing their own health and well‐being. Given the multiple stressors inherent in becoming a health professional, which can only have been exacerbated with the COVID‐19 pandemic, the program was a way of providing students with the tools to survive the practicum experience and become competent healthcare professionals.

## 6 RELEVANCE FOR CLINICAL PRACTICE

This study provides an example of how universities can teach healthcare professional students to recognize and manage stress while on professional practicum. The results confirm it is possible to promote positive behavior change to support resilience and well being in response to stressful clinical environments.

## AUTHOR CONTRIBUTIONS

Study design: Lynda J Ross, Lauren T Williams, Lana J Mitchell, Patrick J Lynch, Jonathan P Munro.

Data collection: Lana J Mitchell.

Data analysis: Lynda J Ross, Lana J Mitchell, Emily C Williams.

Manuscript writing: Lynda J Ross, Lauren T Williams, Emily C Williams, Lana J Mitchell, Patrick J Lynch, Jonathan P Munro.

## CONFLICT OF INTEREST

None declared.

## Data Availability

Data associated with the study are available on written request to the corresponding author.
